# Array CGH data modeling and smoothing in Stationary Wavelet Packet Transform domain

**DOI:** 10.1186/1471-2164-9-S2-S17

**Published:** 2008-09-16

**Authors:** Heng Huang, Nha Nguyen, Soontorn Oraintara, An Vo

**Affiliations:** 1 Department of Computer Science and Engineering, University of Texas at Arlington, TX, USA; 2 Department of Electrical Engineering, University of Texas at Arlington, Texas, USA

## Abstract

**Background:**

Array-based comparative genomic hybridization (array CGH) is a highly efficient technique, allowing the simultaneous measurement of genomic DNA copy number at hundreds or thousands of loci and the reliable detection of local one-copy-level variations. Characterization of these DNA copy number changes is important for both the basic understanding of cancer and its diagnosis. In order to develop effective methods to identify aberration regions from array CGH data, many recent research work focus on both smoothing-based and segmentation-based data processing. In this paper, we propose stationary packet wavelet transform based approach to smooth array CGH data. Our purpose is to remove CGH noise in whole frequency while keeping true signal by using bivariate model.

**Results:**

In both synthetic and real CGH data, Stationary Wavelet Packet Transform (SWPT) is the best wavelet transform to analyze CGH signal in whole frequency. We also introduce a new bivariate shrinkage model which shows the relationship of CGH noisy coefficients of two scales in SWPT. Before smoothing, the symmetric extension is considered as a preprocessing step to save information at the border.

**Conclusion:**

We have designed the SWTP and the SWPT-Bi which are using the stationary wavelet packet transform with the hard thresholding and the new bivariate shrinkage estimator respectively to smooth the array CGH data. We demonstrate the effectiveness of our approach through theoretical and experimental exploration of a set of array CGH data, including both synthetic data and real data. The comparison results show that our method outperforms the previous approaches.

## Background

Gene amplifications or deletions frequently contribute to tumorigenesis. When part or all of a chromosome is amplified or deleted, a change in DNA copy number results. Characterization of these DNA copy number changes is important for both the basic understanding of cancer and its diagnosis. Cancer researchers currently use array comparative genomic hybridization (array CGH) to identify sets of copy number changes associated with the particular cancer or its congenital and developmental disorders. In array CGH, because the clones contain sequences information directly connecting with the genome database, array CGH offers rapid genome-wide analysis at high resolution and the information it provides is directly linked to the physical and genetic maps of the human genome. Bacterial Artificial Chromosomes (BAC) based CGH arrays were amongst the first genomic arrays to be introduced [1] and are routinely used to detect single copy changes in the genome, owing to their high resolution in the order of 1 Mb [1,2]. More recently Oligonucleotide aCGH [3,4] was developed to allow flexibility in probe design, greater coverage, and much higher resolution in the order of 35–100 Kb [5].

In order to develop effective methods to identify aberration regions from array CGH data, the previous research works focus on both smoothing-based [5-9] and segmentation-based data processing [10-14]. The array CGH is very noisy. For example, in cDNA array CGH data, the signal to noise ratio is often approximately 1 (0 dB) [15]. Research in this area has been active in the last few years. Beheshti *et al. *proposed to use the robust locally weighted regression and smoothing scatterplots (lowess) method in [6]. Eilers and Menezes [7] perform a quantile smoothing method based on the minimization of the sum of absolute errors to create sharper boundaries between segments. Hsu *et al. *[8] investigated the usage of maximal overlap discrete wavelet transform (MODWT) in the analysis of array CGH data. They have shown translation invariant wavelets are promising methods for array CGH data smoothing and also observed that the denoising techniques may miss singleton clones that have small changes but somehow are consistent across tumors. In 2005, Lai [16] compared 11 different algorithms for analyzing array CGH data. Many smoothing and estimation methods were included in [16] such as CGHseg (2005) [17], Quantreg (2005) [7], CLAC (2005) [18], GLAD (2004) [11], CBS (2004) [14], HMM (2004) [19], MODWT (2005) [8], Lowess [6], ChARM (2004) [13], GA (2004) [12], ACE (2005) [20]. Lai concluded that Wavelet, Quantreg and Lowess method gave better detection results (higher true position rate and lower false position rate) than other methods. So, the wavelet based smooth was considered as the promising approach. More recently Y. Wang and S. Wang [5] extended the stationary wavelet (SWT) denoising and regression for nonequispaced data, because the physical distance between adjacent probes along a chromosome are not uniform, even vary drastically. However, if a signal is decomposed by using SWT or MODWT, we get unequal sub-bands and a long high frequency sub-bands. Because true CGH signals include many step functions, they contain important information in high frequency. If long high frequency is used to remove noise, maybe, some high frequency true information of CGH will be loosen.

In this paper, we propose to use the Stationary Wavelet Packet Transform (SWPT) to denoise the array CGH data. Because, in SWPT, all sub-bands are also shift invariant, each sub-band provides a shiftable description of signal in a specific scale as the same SWT or MODWT. SWPT analyzes signal to many equally frequency sub-bands. So, information in both of low and high frequency sub-band are saved. Moreover, new bivariate shrinkage function is used in SWPT instead of universal thresholding at the first time, soft thresholding [21-23] and BayesShrink [24]. We demonstrate the effectiveness of our approach through theoretical and experimental exploration of a set of array CGH data, including both synthetic data and real array CGH data. The comparison results show that our method outperforms the previous approaches about 6.4% – 57.9%. Let's see detail results in next section.

## Results and discussion

In this section, results of our proposed methods such as the SWPT and the SWPT-Bi will be compared to the other efficient smooth methods such as the Lowess [16], the Quantreg [7,25], the SWTi [5], the DTCWTi-bi [26]. In our experiments, the artificial chromosomes are generated using the methods proposed in [27] and [5]. Finally, real data examples are showed to make sure that our methods are still better the others.

### Synthetic data

First, we describe how to create synthesis data as follow.

#### Artificial chromosome generation

Willenbrock and Fridlyand [27] proposed a simulation model to create the synthetic array CGH data with equally spaced along the chromosome. More recently Y. Wang and S. Wang [5] extended this model by placing unequally spaced probes along chromosome. As suggested in [27] and [5], the chromosomal segments with DNA copy number *c *= 0, 1, 2, 3, 4 and 5 are generated with probability 0.01, 0.08, 0.81, 0.07, 0.02 and 0.01. The lengths for segments are picked up randomly from the corresponding empirical length distribution given in [27]. Each sample is a mixture of tumor cells and normal cells. A proportion of tumor cells is *P*_*t*_, whose value is from a uniform distribution between 0.3 and 0.7. As in paper [27], the *log*2*ratio *is calculated by

(1) $$log2ratio=\log_{2}\left(\frac{cP_{t}+2(1-P_{t})}{2}\right),$$

where *c *is the assigned copy number. The expected *log*2*ratio *value is then the latent true signal.

Gaussian noises with zero mean and variance $\sigma_{n}^{2}$ are added to the latent true signal. Till now, we get the equally spaced CGH signal. Because the distances between two probes are randomly, the best way to get these distances is from the UCSF HumArray2 BAC array. Thus, we create a real CGH signal from the equally spaced CGH signal when the unequally spaced probes are placed on the chromosome. Now, we have many artificial chromosomes of length 200 *Mbase *which are created by many noise levels *σ*_*n *_= 0.1, 0.125, 0.15, 0.175, 0.2, 0.25 and 0.275.

#### Comparison by RMSE

In this section, we will present the results when applying six methods such as the Lowess [16], the Quantreg [7,25], the SWTi [5], the DTCWTi-bi [26] and our methods the SWPT and the SWPT-Bi. One thousand artificial chromosomes with seven different noise levels *σ*_*n *_= 0.1, 0.125, 0.15, 0.175, 0.2, 0.25 and 0.275 are denoised.

**Figure 1:**
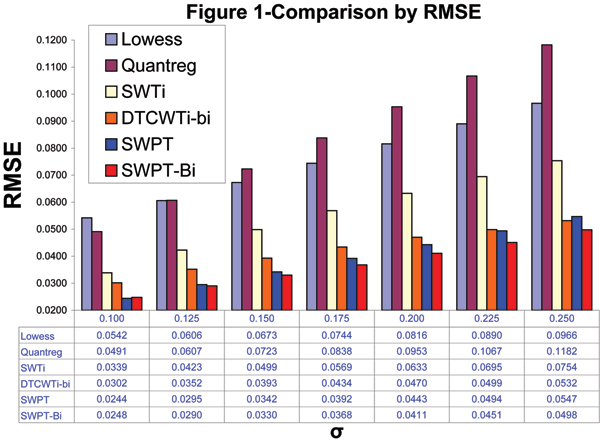
**Comparison by RMSE**. Comparison of average RMSEs obtained from the 1,000 artificial chromosomes with each of the 7 noise levels using the Lowess, the Quantreg, the SWTi, the DTCWTi-bi and our methods such as the SWPT and the SWPT-Bi.

The denoising results of all methods are shown in the Figure 1. We can see that the proposed SWPT and SWPT-Bi methods yield the better performance than the others. The SWPT and SWPT-Bi outperform the Lowess by 43.4% – 55% and 48.4% – 54.2% respectably, the Quantreg by 50.3% – 53.7% and 49.5% – 57.9% respectably and the SWTi by 27.5% – 31.5% and 26.8% – 35.3% in terms of the root mean squared errors (RMSEs). If compared the DTCWTi-bi, the SWPT-Bi gets better by 6.4% – 17.9% for seven noise level and the SWPT performs better by 1% – 19.2% for six noise levels (0.1 – 0.225). For all noise levels, the SWPT-Bi consistently achieves much better results than the others.

**Figure 2:**
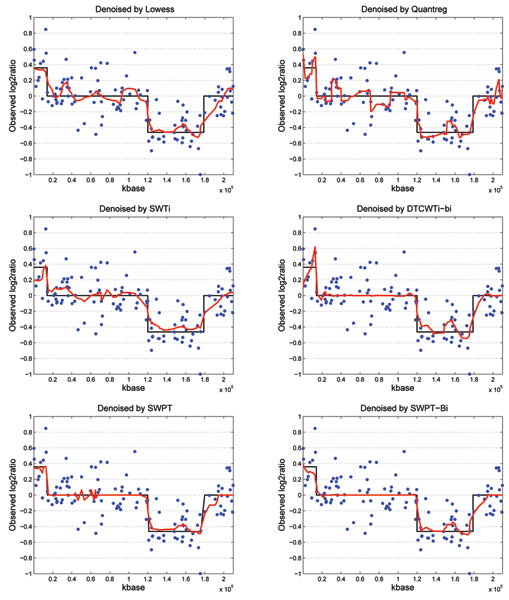
**Example of wavelet denoising results**. Example of wavelet denoising results at the noise level of *σ *= 0.2 using the Lowess, the Quantreg, the SWTi, the DTCWTi-bi and our methods such as the SWPT and the SWPT-Bi.

Some examples of wavelet denoising results by using the Lowess, the Quantreg, the SWTi, the DTCWTi-bi, the SWPT and the SWPT-Bi methods are shown in Figure 2 at the noise level of *σ *= 0.2. In those Figures, the black solid lines represent the latent true signals, the blue points stand for the noisy DNA copy data *log*_2_*ratio *at the probe loci and the red lines correspond to the denoised data. We should note that the line connecting the denoised data points is only for visualization purpose.

At the copy three *c *= 3 (from 1 *kbase *to 1.4 × 10^4 ^*kbase*) as shown in Figure 2, the *log*_2_*ratio *value of the latent true signal is 0.3598, but these values of the Quantreg, the SWTi and the DTCWTi-bi based denoised signal in Figure 2 are from 0.2262 to 0.4966, from 0.1774 to 0.3828 and from 0.09233 to 0.6182 respectably. These values can cause a mistake when we segment the DNA copy number data. However, the denoised data using the Lowess, the SWPT and the SWPT-Bi will be segmented correctly as the copy three (from 0.2 to 0.4) because the *log*_2_*ratio *values are from 0.2129 to 0.3619, 0.2794 to 0.3649 and from 0.2565 to 0.3964. At the copy two *c *= 2 (from 1.4 × 10^4 ^*kbase *to 1.2 × 10^5 ^*kbase*), the denoised data in the second sub-figure (denoised by Quantreg) of Figure 2 has an amplitude of 0.2262 which will make an error in segmentation process, while the denoised data in other sub-figures of Figure 2 will give a correct segmentation. In this copy, the denoised signals using DTCWTi-bi, the SWPT and SWPT-Bi are approximately the latent true signals, while the denoised data using the Lowess, the Quantreg and the SWTi have many ripples. At the copy zero *c *= 0 (from 1.2 × 10^5 ^*kbase *to 1.79 × 10^5 ^*kbase*), if we use TPR (true position rate = number of denoised probes belove -0.4/total of true probes), the Lowess, the Quantreg and our methods gave a ratio of 22 over 34 instead of 17/34 and 14/34 of the SWTi and DTCWTi-bi. However, the denoised signals of the Lowess, the SWPT and the SWPT-Bi look better than of the Quantreg. At the copy two *c *= 2 (form 1.79 × 10^5 ^*kbase *to the end of the chromosome), the fourth, fifth and sixth sub-figures's signal (denoised by DTCWTi-bi, SWPT the and SWPT-Bi) of Figure 2 look smoother than the others. Furthermore, the denoised signals at the first sub-figure (the Lowess's) and the second sub-figure (the Quantreg's) may cause error when segmentation because denoised signals change from -0.3133 to 0.101 (Lowess) and from -0.2119 to 0.2084 (Quantreg).

From above results, we can see that our proposed SWPT and SWPT-Bi methods with the stationary wavelet packet transform are better than the others. Now, real data will be used to test five smoothing methods as follow.

### Real data examples

**Figure 3:**
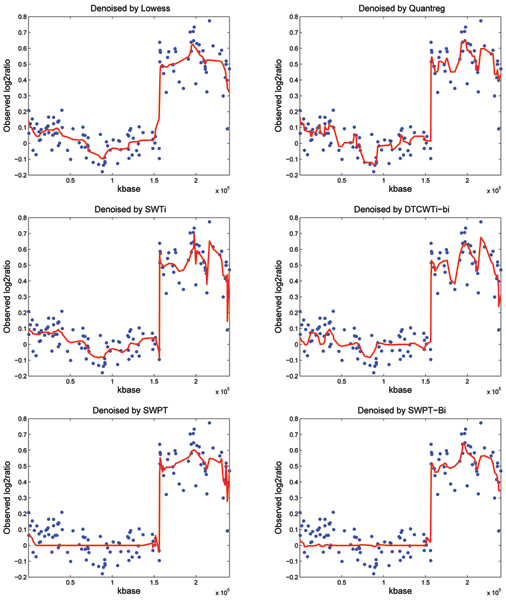
**Real Data Examples**. The wavelet denoising results of array CGH data on chromosome 1 in the real signal GM13330 using some methods such as the Lowess, the Quantreg, the SWTi, the DTCWTi-bi and our methods such as the SWPT and the SWPT-Bi.

In this paper, the BAC array data on 15 fibroblast cell lines [8,28] has been used to show that denoising by the SWPT and the SWPT-Bi are better than by the others such as the Lowess, the Quantreg, the SWTi and the DTCWTi-bi. This data set is from Stanford University, which can be freely downloaded at [29]. Because the true copy number changes are known for these cell lines, we choose these data as a proof of principles. We pick up the chromosome 1 of GM13330 from these data and apply six algorithms for denoising. In Figure 3, the number copies are two and four. At the copy two (from 1 *kbase *to 1.56 × 10^5 ^*kbase*), the SWPT and SWPT-Bi based smoothed signals are smoother than the others. With the copy four, from 1.56 × 10^5 ^*kbase *to the end of this chromosome, the performance of the Lowess, the SWPT and the SWPT-Bi based denoising methods are the better than of the Quantreg, the SWTi and the DTCWTi-bi. From the above figures, we can believe that our methods perform better than the others in denoising of real CGH data.

## Conclusion

In this paper, we explored the stationary wavelet packet transform method with the new bivariate shrinkage estimator in array CGH data denoising study. In the simulation situations, the denoising results from the SWPT and the SWPT-Bi are much better (improve 6.4% – 57.9%) than the previous methods in terms of the root mean squared error measurement at different noise levels. Furthermore, we also demonstrate our method by using the real array CGH data. In our future work, we will develop a smoothing and segmentation combinatorial algorithm to improve the aberration regions identification from DNA copy number data.

## Methods

Our methods named the SWPT (SWPT and universal shrinkage function) and the SWPT-Bi (SWPT and bivariate shrinkage function) will be introduced. First, lets review wavelet transform and see how SWPT operates.

### Wavelet methods

We will provide a brief review of wavelet transforms which were used for array CGH data smoothing and is used by this paper. We should note that the simple wavelet transform will be introduced firstly and the SWPT will be mentioned finally.

#### Discrete wavelet transform

**Figure 4:**
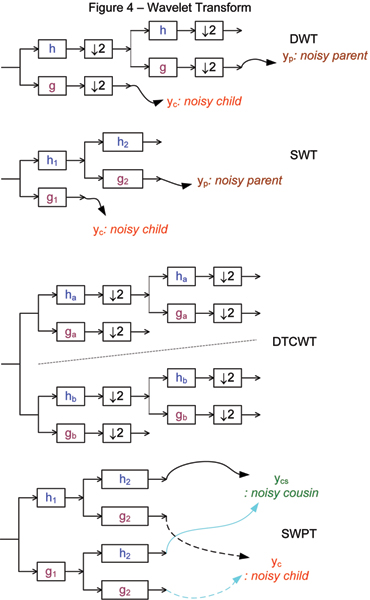
**Wavelet Transform**. Analysis filter bank and the position of child, parent and cousin coefficients of discrete wavelet transform (DWT), stationary wavelet transform (SWT), dual tree wavelet complex transform (DTCWT), discrete wavelet packet transform (DWPT) and stationary wavelet packet transform (SWPT).

The discrete wavelet transform (DWT), showed in Figure 4, based on the octave band tree structure, can be viewed as the multiresolution decomposition of a signal. It takes a length *N *sequence, and generates an output sequence of length *N *using a set of lowpass and highpass fiters followed by a decimator. It has *N/*2 values at the highest resolution, *N/*4 values at the next resolution, and *N/*2^*L *^at the level *L*. Because of decimation, the DWT is a critically sampled decomposition. However, the drawback of DWT is the shift variant property. In signal denoising, the DWT creates artifacts around the discontinuities of the input signal [30]. These artifacts degrade the performance of the threshold-based denoising algorithm.

#### Stationary wavelet transform

The stationary wavelet transform (SWT) [30], showed in Figure 4, is similar to the DWT except that it does not employ a decimator after filtering, and each level's filters are upsampled versions of the previous ones. The SWT is also known as the shift invariant DWT. The absence of a decimator leads to a full rate decomposition. Each subband contains the same number of samples as the input. So for a decomposition of *L *levels, there is a redundant ratio of (*L *+ 1): 1. However, the shift invariant property of the SWT makes it preferable for the usage in various signal processing applications such as denoising and classification because it relies heavily on spatial information. It has been shown that many of the artifacts could be suppressed by a redundant representation of the signal [30].

#### Dual-tree complex wavelet transform

A dual-tree structure that produces a dyadic complex DWT, showed in Figure 4, is proposed by Kingsbury [31,32]. In the case of 1-D signals, the structure consists of two binary trees of multi-resolution decomposition of the same signal. It is therefore an overcomplete representation with a redundant ratio of 2:1. In the two trees, the filters are designed in such a way that the aliasing in one branch in the first tree is approximately canceled by the corresponding branch in the second tree. The relation between the wavelet filters of the two trees yields shift-invariant property [31].

The analysis FB for the DTCWT is an iterative multi-scale FB. Each resolution level consists of a pair of two-channel FBs. The purpose of the dual-tree CWT is to provide a shiftable and scalable multiresolution decomposition. The input signal is passed through the first level of a multiresolution FB. The low frequency component, after decimation by 2, is fed into the second level decomposition for the second resolution. The outputs of the two trees are the real and imaginary parts of complex-valued subbands. To reconstruct the signal, the real part and imaginary part are inverted to obtain two real signals, respectively. These two real signals are then averaged to obtained the final output. For more details of the construction of the dual-tree, the reader is referred to [33].

#### Discrete wavelet packet transform

We continue with an another basic othornormal wavelet transform. Discrete wavelet packet transform (DWPT), which can be readily computed by using a very simple adjustment of the pyramid algorithm for DWT, will be mentioned. All of DWPT scales are performed at the same level *j*. The *jth *level DWPT decomposes the frequency interval [0, 1/2] into 2^*j *^equal and individual intervals, each of which has *N/*2^*j *^values if taking a length *N *sequence. DWPT still keeps a shift variant property.

#### Stationary wavelet packet transform

Stationary Wavelet Packet Transform (SWPT), showed in Figure 4, still keeps two important properties of SWT such as shift invariance and redundancy. In the SWPT, both scaling and wavelet coefficients are subjected to the high-pass and low-pass filter when computing the next level coefficients. At the given level *L*, there are 2^*L *^scales with the same length as the input signal's. The redundant ratio is (2^*L*^): 1 for a decomposition of *L *levels. SWPT is really combination of SWT and DWPT. So, it is very useful in denoising of DCN data. After wavelet transform, reader should be introduced a new shrinkage function to remove noise of CGH data in SWPT domain as follows.

### New vivariate shrinkage function for SWPT-based denoising

In this sub-section, the bivariate shrinkage function which describes the relationship of child and parent (Figure 4) coefficients will be reminded. Because SWPT, which decomposes a signal into many subbands at the same scale, just has child and cousin coefficients (Figure 4) at the same level, new bivariate shrinkage function will be developed to exploit the relationship between child and cousin coefficients. A simple denoising algorithm via wavelet transform consists of three steps: decompose the noisy signal by wavelet transform, denoise the noisy wavelet coefficients according to some rules and take the inverse wavelet transform from the denoised coefficients. To estimate wavelet coefficients, some of the most well-known rules are universal thresholding, soft thresholding [21-23] and BayesShrink [24]. In these algorithms, the authors assumed that wavelet coefficients are independent. Sendur and Selesnick [34] has recently exploited the dependency between coefficients and proposed a non-Gaussian bivariate pdf for the child coefficient *w*_*c *_and its parent *w*_*p*_. Nguyen *et el *[26,35] applied that function to recover CGH data successfully and got some promising results.

Now basing on the idea in [34], we try to discover the connection of child and cousin coefficients in SWPT with CGH data. We assume that we get the DNA copy number data *Y *which includes the deterministic signal *D *and the independent and identically distributed (IID) Gaussian noise *n*. This Gaussian noise has zero mean and variance $\sigma_{n}^{2}$.

(2) *Y *= *D *+ *n*.

After decomposing the data *Y *by the SWPT, we get the coefficients **y**_*k*_. In the wavelet domain, those coefficients can be formulated as

(3) $$\begin{array}{l} y_{1}=w_{1}+n_{1},\\ y_{2}=w_{2}+n_{2}, \end{array}$$

where *y*_1 _and *y*_2 _are noisy wavelet coefficients, *w*_1 _and *w*_2 _are true coefficients, *w*_2 _represents the cousin of *w*_1 _(child), *n*_1 _and *n*_2 _are independent Gaussian noise coefficients. If the cousin scale *y*_2 _continue being decomposed, we will get detail and approximation coefficients. Let's call *y*_3 _as approximation coefficients of *y*_2_. We can calculate *y*_3 _from *y*_2 _by the follow equation:

(4) $$\begin{array}{lll} y_{3} & = & w_{3}+n_{3},\\ y_{3}[n] & = & h[n]*y_{2}[n]\\ & = & \sum_{k=1}^{N}\left(h[n-k].y_{2}[k\right]), \end{array}$$

where *h *[*n*] is the low pass filter and *N *is the length of signal *y*_2_. In general, we can write

(5) **y **= **w **+ **n**,

where **y **= (*y*_1_, *y*_3_), **w **= (*w*_1_, *w*_3_) and **n **= (*n*_1_, *n*_3_). The noise pdf of two next scales should be followed as

(6) $$p_{n}(n)=\frac{1}{2\pi \sigma_{n}^{2}}\exp(-\frac{n_{1}^{2}+n_{3}^{2}}{2\sigma_{n}^{2}}).$$

The standard MAP estimator [34] of **w **from **y **is followed as

(7) $$\hat{w}(y)=\arg\underset{w}{\max}[\log(p_{n}(y\text{-}w))+\log(p_{w}(w))].$$

As [34], we propose a non-gaussian bivariate pdf for *w*_1 _and *w*_3 _as

(8) $$p_{w}(w)=\frac{k}{2\pi \sigma^{2}}\exp(-\frac{\sqrt{2k}}{\sigma }\sqrt{|w_{1}{|}^{2}+|w_{3}{|}^{2}}).$$

With this pdf, two variables *w*_1 _and *w*_3 _are really dependent. Let us define:

(9) $$\begin{array}{lll} f(w) & = & \log(P_{w}(w))\\ & = & \log(\frac{k}{2\pi \sigma^{2}})-\frac{\sqrt{2k}}{\sigma }\sqrt{|w_{1}{|}^{2}+|w_{3}{|}^{2}}. \end{array}$$

By using (6), (7) becomes:

(10) $$\hat{w}(y)=\arg\underset{w}{\max}[-\frac{{\left(y_{1}-w_{1}\right)}^{2}+{\left(y_{3}-w_{3}\right)}^{2}}{2\sigma_{n}^{2}}+f(w)].$$

Solving above equation is the same solving of two following equations:

(11) $$\frac{\left(y_{1}-w_{1}\right)}{\sigma_{n}^{2}}+f_{w_{1}}(\hat{w})=0,$$

(12) $$\frac{\left(y_{3}-w_{3}\right)}{\sigma_{n}^{2}}+f_{w_{3}}(\hat{w})=0,$$

where $f_{w_{1}}$ and $f_{w_{3}}$ represent the derivative of *f*(*w*) with respect to *w*_1 _and *w*_3_, respectively. We can get $f_{w_{1}}$ and $f_{w_{3}}$ from (9)

(13) $$f_{w_{1}}(\hat{w})=\frac{\sqrt{2k}w_{1}}{\sigma \sqrt{|w_{1}{|}^{2}+|w_{3}{|}^{2}}}.$$

(14) $$f_{w_{3}}(\hat{w})=\frac{\sqrt{2k}w_{3}}{\sigma \sqrt{|w_{1}{|}^{2}+|w_{3}{|}^{2}}}.$$

Substituting (13) and (14) into the (11) and (12) gives:

(15) $$\begin{array}{cc} \hat{w_{1}}\cdot (1+\frac{\sqrt{2k}\sigma_{n}^{2}}{\sigma r})=y_{1}, & \hat{w_{3}}\cdot (1+\frac{\sqrt{2k}\sigma_{n}^{2}}{\sigma r})=y_{3}, \end{array}$$

where $r=\sqrt{|\hat{w_{1}}{|}^{2}+|\hat{w_{3}}{|}^{2}}$. Drawing *r *from (15):

(16) $$r={\left(\sqrt{|y_{1}{|}^{2}+|y_{3}{|}^{2}}-\frac{\sqrt{2k}\sigma_{n}^{2}}{\sigma }\right)}_{+}.$$

If replacing *r *by (16) into (15), the MAP estimator can be written as:

(17) $${\hat{w}}_{1}=\frac{{\left(\sqrt{|y_{1}{|}^{2}+|y_{3}{|}^{2}}-\frac{\sqrt{2k}\sigma_{n}^{2}}{\sigma }\right)}_{+}}{\sqrt{|y_{1}{|}^{2}+|y_{3}{|}^{2}}}\cdot y_{1},$$

where (*u*)_+ _is defined by

(18) $${\left(u\right)}_{+}=\{\begin{array}{cc} 0 & \text{if }u<0,\\ u & \text{otherwise}\text{.} \end{array}$$

Replacing *y*_3 _from (4) to (17), we can rewrite the MAP estimator as

(19) $${\hat{w}}_{1}=\frac{y_{1}{\left(\sqrt{|y_{1}{|}^{2}+|\sum_{k=1}^{N}(h[n-k]y_{2}[k]){|}^{2}}-\frac{\sqrt{2k}\sigma_{n}^{2}}{\sigma }\right)}_{+}}{\sqrt{|y_{1}{|}^{2}+|\sum_{k=1}^{N}(g[n-k].y_{2}[k]){|}^{2}}}$$

In (19), *σ *can be estimated by

(20) $$\hat{\sigma }=\sqrt{{\left({\hat{\sigma }}_{y}^{2}-{\hat{\sigma }}_{n}^{2}\right)}_{+}},$$

where ${\hat{\sigma }}_{n}$ is the noise deviation which is estimated from the finest scale wavelet coefficients by using a robust median estimator [22] as follows

(21) $${\hat{\sigma }}_{n}^{2}=\frac{median(|y_{i}|)}{0.6745}.$$

${\hat{\sigma }}_{y}$ is the deviation of observation signal estimated by

(22) $${\hat{\sigma }}_{y}^{2}=\frac{1}{M}\sum_{y_{i}\in N(k)}|y_{i}{|}^{2},$$

where *M *is the size of the neighborhood *N*(*k*). In the packet wavelet transform, the cousin scales have not any parent scale. In this case, we can use hard thresholding estimator [21] to recover cousin coefficients ${\hat{w}}_{cs}$:

(23) $${\hat{w}}_{cs}={\left(y_{cs}-\sigma_{n}\sqrt{2\log N}\right)}_{+},$$

Now, after getting new bivariate shrinkage functions, we should compare this new function to the bivariate function of Sendur [34] as the table 1. From this table, our function has four different parts with Sendur's. Now, we have one more pre-processing step to save data at the border of CGH data. That is signal extension which will be discussed more as follow.

**Table 1:** Comparison table of our new bivariate shrinkage function and function in [34]

Comparison Table		
Method	New bivariate shrinkage function.	Bivariate shrinkage function in [34].

Applying to Relationship *y*_3_ Transform	CGH data. child and cousin coefficient. *y*_3 _= h * *y*_2_, where h is a low pass filter. SWPT and DWPT.	image. child and parent coefficient. *y*_*p *_= g * *y*_2_, where g is a high pass filter. DWT, SWT and DTCWT.

### Signal extension

CGH data is finite signal. If we apply wavelet smooth method directly, we may get error at the border of denoised signal. So, extension step is a very important preprocessing step before denoising. There are three main extension methods. According to the book [36] (chapter 8), symmetric extension is the best if applied to a filtered image because we can save information at the border better. With CGH data, we also need save the information at the border. So, we recommend that symmetric extension method should be used as a preprocessing step before denoising. Let's assume that the length of the CGH signal is *N*. In order to get the best performance in the wavelet denoising algorithm, the length of the input signal is required to be a power of two [37]. If *N *is not a power of two, we can extend our signal to make sure *N *= 2^*j *^by using symmetric extension method. Finally, the SWPT-Bi will be detailed in next part.

### Proposed method

The DWT with the redundant ratio of 1:1 is efficient for the denoising applications. However, the DWT creates artifacts around the discontinuities of the input signal [30] because it is shift-variant. To overcome this problem, SWT [5] or MODWT [8] and DTCWT [26,35] with translation invariant property was proposed for signal denoising. It has been shown that many of the artifacts could be suppressed by a redundant representation of the signal [30]. One important thing is that CGH data contains many step functions which their information is in both low frequency and high frequency. The above wavelet methods have one disadvantage which some high frequency components of CGH data were removed. In this paper, the SWPT will be used to overcome some above problems because it keeps shift invariant property and looks for signal both in low frequency and in high frequency band for denoising operation. Several methods were proposed for selecting thresholding values such as hard universal [21,22] and un-universal thresholding [23]. However, the dependency between wavelet coefficients are not exploited in these methods. Thus, we propose the usage of shift invariant SWPT and new bivariate shrinkage estimator which takes advantage of the dependency between wavelet coefficient and its cousin for array-based DNA copy number data denoising.

Our purpose is to find $\hat{D}$ from *Y *so that the root mean squared error (RMSE) (24) is the smallest.

(24) $$RMSE=\sqrt{\frac{1}{N}\sum_{i}^{N}{\left({\hat{D}}_{i}-D_{i}\right)}^{2}},$$

and *N *is the number of input samples, *D *= {*D*_*i*_} and $\hat{D}=\{{\hat{D}}_{i}\}$.

We propose a stationary wavelet packet transform and new bivariate shrinkage function based smooth method (SWPT-Bi). The SWPT-Bi can be summarized as follows:

**Step 1**: *Extend Y by using symmetric extension method and decompose new data Y' by the SWPT to L levels as (25). The numbers of decomposition levels *[38]*(at the remark *11) *can be computed by*

(25) *L *= log_2_(*N*) - *J*.

*where J *= 3, 4, 5, 6. *This is a perfect number of levels *[38]*which yields the best denoising result. In this paper, we use J *= 4 *as the same in *[8]*and *[5].

**Step 2**: *Calculate the noise variance *${\hat{\sigma }}_{n}^{2}$*and the marginal variance *${\hat{\sigma }}^{2}$*for wavelet coefficient y*_*k *_*by using (21), (22) and (20)*.

**Step 3**: *Estimate the child coefficients *${\hat{w}}_{c}={\hat{w}}_{1}$*as in (19) and estimate the counsin coefficients *${\hat{w}}_{cs}$*as in (23). In this case, k *= 1.45 *should be chosen*.

**Step 4**: *Reconstruct data *$\hat{D}$*from the denoised coefficients *${\hat{w}}_{c}$*and *${\hat{w}}_{cs}$*by taking inverse SWPT*.

We also propose one simple method SWPT. In the SWPT method, hard thresholding [22] method is used. The SWPT method can be summarized as follows:

**Step 1**: *Extend Y by using symmetric extension and decompose new data using the SWPT*.

**Step 2**: *Estimate the noise variance *$\sigma_{n}^{2}$*by (21)*.

**Step 3**: *Find the denoised coefficients from noisy coefficients as follow*

(26) $${\hat{w}}_{i}={\left(y_{i}-\sigma_{n}\sqrt{2\log N}\right)}_{+},$$

*where N is length of y*.

**Step 4**: *Reconstruct data *$\hat{D}$*from the denoised coefficients *${\hat{w}}_{i}$*by taking inverse SWPT*.

## Competing interests

The authors declare that they have no competing interests.

## Authors' contributions

HH and NN developed the theory, performed the experiments, and wrote the manuscript. SO and AV gave important discussion and contributed to the manuscript. All authors read and approved the final manuscript.
